# Risk factors for subjective and objective cognitive decline in sexual minority older adults

**DOI:** 10.1002/alz.087539

**Published:** 2025-01-09

**Authors:** Riccardo Manca, Jason D. Flatt, Annalena Venneri

**Affiliations:** ^1^ Brunel University London, Uxbridge United Kingdom; ^2^ University of Nevada Las Vegas School of Public Health, Las Vegas, NV USA; ^3^ Brunel University London, London United Kingdom; ^4^ University of Parma, Parma Italy

## Abstract

**Background:**

Sexual minority older adults (SMOAs) report subjective cognitive decline (SCD) more than heterosexual older adults (HOAs). Inconsistent findings have emerged about the risk of cognitive decline in SMOAs. This study aimed to compare the impact of multiple psycho‐social risk factors on both objectively assessed and subjectively reported cognitive decline between HOAs and SMOAs.

**Method:**

Two samples of self‐identified HOAs (*n* = 4180) and SMOAs (*n* = 92) who completed Waves 8 and 9 of the English Longitudinal Study of Ageing were selected. Reliable change indices for episodic and semantic memory were created to assess longitudinal cognitive changes. SCD was self‐assessed for both memory and general cognition. Mental and cardiovascular health, marital status, loneliness, and socio‐economic status (SES) were investigated as risk factors for cognitive decline. The effect of each risk factor and their interaction effects with sexual orientation on memory changes and SCD were tested, respectively, with general linear and logistic regression models. Age, education, and sex were included as covariates. A sensitivity analysis was carried out in a sub‐sample (*n* = 92, per group) matched for demographics.

**Result:**

Cognitive changes and rates of SCD were similar between SMOAs and HOAs. All risk factors, apart from marital status, were associated SCD risk, while only SES was associated with decline in episodic memory (β = .01, *p* = .038). A SES×sexual orientation interaction was found for semantic memory (β = ‐.06, *p* = .042); among those in the lowest quintile, SMOAs had better semantic memory preservation over time than HOAs. A sensitivity analysis found among those in the highest quintile, SMOAs had worse semantic memory decline than HOAs.

**Conclusion:**

SCD was associated with a multitude of risk factors, in line with previous evidence of SCD as a heterogeneous construct. This was similar between SMOAs and HOAs. Longitudinal changes in semantic memory were, instead, differentially impacted by SES in the groups. Sensitivity analysis confirmed a potential lack of protective effect in SMOAs, although the groups did not differ for SES. These findings may be interpreted within the framework of material deprivation, which is more likely among SMOAs and may contribute to their cognitive decline more severely than in HOAs.

